# Symmetrically Ion‐Gated In‐Plane Metal‐Oxide Transistors for Highly Sensitive and Low‐Voltage Driven Bioelectronics

**DOI:** 10.1002/advs.202103275

**Published:** 2022-03-03

**Authors:** Jingu Kang, Young‐Woo Jang, Sang Hee Moon, Youngjin Kang, Jaehyun Kim, Yong‐Hoon Kim, Sung Kyu Park

**Affiliations:** ^1^ School of Electrical and Electronics Engineering Chung‐Ang University Seoul 06974 Korea; ^2^ School of Advanced Materials Science and Engineering Sungkyunkwan University Suwon 16419 Korea; ^3^ Department of Chemistry and Materials Research Center Northwestern University 2145 Sheridan Road Evanston IL 60208 USA

**Keywords:** amorphous oxide semiconductors, electric double layer, ion‐gel, monolithic passivation, symmetrically gated structure, thin‐film transistor

## Abstract

To provide a unique opportunity for on‐chip scaled bioelectronics, a symmetrically gated metal‐oxide electric double layer transistor (EDLT) with ion‐gel (IG) gate dielectric and simple in‐plane Corbino electrode architecture is proposed. Using amorphous indium‐gallium‐zinc oxide (*a*‐IGZO) semiconductor and IG dielectric layers, low‐voltage driven EDLTs with high ionotronic effects can be realized. More importantly, in contrast to the conventional asymmetric rectangular EDLTs which can cause non‐uniform potential variation in the active channel layer and eventually degrade the sensing performance, the new symmetrical in‐plane type EDLTs achieve high and spatially uniform ion responsive behaviors. The symmetrically gated *a*‐IGZO EDLTs exhibited a responsivity of 129.4% to 5 ppm mercury (Hg^2+^) ions which are approximately three times higher than that with conventional electrode structure (responsivity of 38.5%). To confirm the viability of the new device architectures and the findings, the detailed mechanism of the symmetric gating effects in the in‐plane EDLTs with a variety of electrical characterization and 3D fine element analysis simulations is also discussed.

## Graphical abstract

A symmetrically gated metal oxide electric double layer transistor (EDLT) with ion‐gel dielectric and simple in‐plane Corbino electrode architecture for on‐chip scaled bioelectronics, including advantages of the symmetric structure for uniform potential variation in the active channel layer, is investigated. The corbino‐type EDLT exhibits approximately three times higher responsivity to mercury ions compared to the conventional rectangular‐type electrode structure.

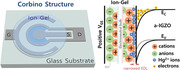

## 1 Introduction

Bioelectronic devices provide a unique opportunity in monitoring the biological signals and diagnosing human diseases.^[^ ^1^, ^2^, ^3^, ^4^, ^5^, ^6^, ^7^ ^]^ Among various candidates for bio‐electronic devices, electrolyte gated transistors are of significant interest due to their facile implementation as building blocks in integrated bioelectronics which can enable the functions such as detection of biological signals and delivery of electrochemical stimulations.^[^ ^8^, ^9^, ^10^, ^11^ ^]^ In typical electrolyte gated transistors, the semiconducting channel layer is surrounded by an ion‐sensitive electrolyte which acts as an ion‐to‐electron conversion layer. Also, a reference electrode is used to apply a gate bias to the electrolyte, controlling the sensing properties. When the variation of ion concentration occurs between the gate electrode and electrolyte, the effective gate bias is changed, resulting in a modulation of output signals. However, in conventional electrolyte gated transistors, the electrolyte and gate electrode are used as integral components,^[^ ^12^, ^13^ ^]^ limiting independent gate‐field control for the operation of individual devices and constraining as an integrated functional building block such as circuit components and stimuli carrying devices. As a result, there has been a growing demand for the development of electrolyte gated transistors capable of independent gate‐field control for integrated functional building blocks in advanced bioelectronics.

Recently, ion‐gel (IG)‐gated transistors have been exploited in various electronic devices such as thin‐film transistors,^[^ ^14^ ^]^ neuromorphic devices,^[^ ^15^, ^16^ ^]^ pressure sensors,^[^ ^17^, ^18^ ^]^ and chemical sensors.^[^ ^19^ ^]^ Especially, the IG film exhibits interesting electrostatic properties such as the formation of high‐capacitance electric double layer (EDL). More importantly, the IG can be physically isolated by using CMOS‐compatible photolithography process, forming an independent structure with adjacent devices. Therefore, using IG film, electric double layer transistors (EDLTs) with high ionotronic effects can be realized with site‐specific input gate biasing. In addition, due to the high‐capacitance of IG film, a high carrier density can be induced in the active channel, which results in high sensitivity and fast response time at a sufficiently low operating voltage. Additionally, the long‐range polarizability of ions and facile processability of IG film allow more intriguing device architectures, simplifying the device geometry.^[^ ^20^ ^]^ For instance, an in‐plane type transistor can be realized where the gate and the source/drain (G/S/D) electrodes lie in the same plane, contributing to reduced fabrication process and enlarged interaction area with the target species. Previously, we demonstrated an IG‐gated in‐plane EDLT operating at a frequency range from static to around 300 Hz.^[^ ^21^ ^]^ With its simple device architecture and high carrier mobility using the metal‐oxide semiconductor channel, the IG‐gated in‐plane EDLT can be a promising platform to realize robust EDLTs for integrated bioelectronics.

In the IG film, however, various intrinsic impurities are present, such as the unreacted starting materials (e.g., chloride), water, polymeric components, and dissolved gases (e.g., oxygen), interfering with the formation of narrow EDLs at each interface. This, indeed, can cause nonuniform potential variation in the active channel layer and degrade the sensing performance, especially when an asymmetric rectangular geometry is used for the in‐plane EDLTs. In addition, this nonuniform gating effect inherently results in a strong dependence of ion sensitivity on applied gate bias and the distance between the gate electrode and the active channel.^[^ ^22^ ^]^ Therefore, to achieve highly and spatially uniform ion responsive behaviors in in‐plane type EDLTs, a new device architecture is now demanded particularly for bio‐sensing applications.^[^ ^22^ ^]^

Herein, we demonstrate an in‐plane type IG‐gated EDLT with Corbino electrode structure, enabling symmetric gating effect for high sensitivity and uniform electrostatic responses to target analytes. The amorphous indium‐gallium‐zinc oxide (*a*‐IGZO)‐based EDLTs exhibited a responsivity of 129.4% to 5 ppm mercury (Hg^2+^) ions, which is approximately three times higher than that with conventional electrode structure (responsivity of 38.5%). To validate the proposed device characteristics, we discuss a more detailed mechanism of the symmetric gating effects and their highly electrostatic responsivity in the in‐plane EDLTs along with an array of electrochemical characterizations and position‐dependent multi‐dimensional fine element analysis (FEA) simulations. We envision that the proposed EDLT with symmetrically gated in‐plane architecture could enable high‐performance and accurate monitoring of biological signals with marginal complexity, offering compatibility with standard CMOS processing and large‐scaled on‐chip device applications.

## 2 Results and Discussion

**Figure 1:**
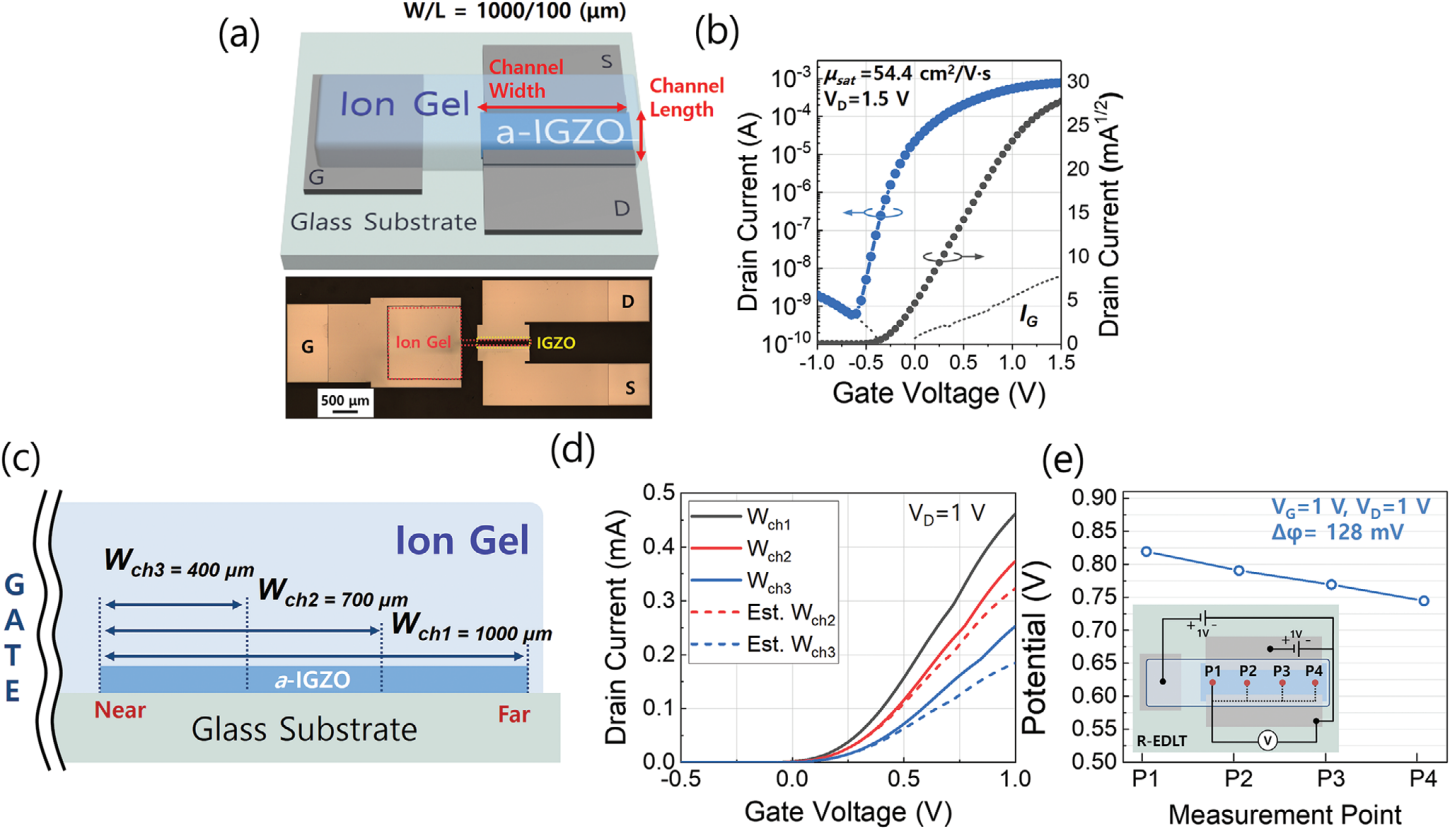
a) The device structure of an R‐EDLT and its optical microscopic (OM) image. b) A transfer characteristic of an R‐EDLT. The gate voltage was swept from −1  to 1.5 V with a step of 25 mV while the drain voltage of 1.5 V is applied to the device. The threshold voltage was −0.2 V and the average saturation mobility of five different R‐EDLTs was 54.4 cm^2^ V^−1^·s ^−1^ with a standard deviation (SD) of 0.785. The dotted line represents gate leakage current. c) Schematic diagram of the channel width variations. The widths of *W*_ch1_, *W*_ch2_, and *W*_ch3_ are 1000, 700, and 400 µm, respectively. d) Transfer characteristics of R‐EDLTs with different channel widths. The colored solid lines represent experimentally measured current characteristics. The colored dashed lines represent estimated drain currents (Est. *I*_D_(*W*_ch2_) = *I*_D_(*W*_ch1_) × 0.7, Est. *I*_D_(*W*_ch3_) = *I*_D_(*W*_ch1_) × 0.4). e) The potential profile of the IG gate dielectric along with the channel width direction under *V*_G_ = *V*_D_ = 1 V. Each point data are the representative of five repeated measurements with an average SD of 0.0039. The inset shows the measurement points of the potential.

The device structure of rectangular‐shaped in‐plane EDLTs (R‐EDLTs) is depicted in **Figure** **1**a. The R‐EDLTs adopted the in‐plane‐gate geometry where the gate electrode is located on the same plane with the *a*‐IGZO channel layer. Using this structure, a side gating operation is possible since the IG gate dielectric is formed over the gate electrode and the *a*‐IGZO channel layer. In typical IG‐gated transistors, the direct contact between the IG gate dielectric and the electrodes causes a substantially high leakage current, which significantly reduces the current on/off ratio. Therefore, to minimize the contact‐induced leakage current and to increase the signal‐to‐noise ratio, a thin monolithic Al_2_O_3_ layer was formed on the surface of Al G/S/D electrodes by photochemical surface modification process prior to depositing the IG gate dielectric.^[^ ^21^ ^]^ Figure S1a, Supporting Information, shows a cross‐section diagram of R‐EDLT and its SEM image. However, the overlap area is still large because the contact area of G/S/D electrodes and the IG dielectric can be considered as the overlap area. Therefore, the S/D electrodes were covered with a 3‐µm‐thick SU‐8 layer as illustrated in Figure S1a, Supporting Information. Also, it was inevitable that there would be polymeric gaps as depicted. In previous studies, the local gate structure that has offsets of a few micro‐meters between G and S/D has been employed as a strategy to reduce overlap capacitance and leakage current.^[^ ^23^, ^24^, ^25^ ^]^ Although electrical performances, such as threshold voltage and saturation mobility, were varied as a function of the offset length, the local gate devices still showed high‐performance transfer characteristics even at a low drain voltage of *V*_D_ = 0.1 V thanks to carrier diffusion over the offset.^[^ ^26^ ^]^ Accordingly, it is quite acceptable to utilize thick polymeric passivation over S/D and thin Al2O3 passivation over gate electrodes in R‐EDLT, and C‐EDLT. Therefore, the thick passivation is beneficial for EDLTs to reduce unintentional elements more such as parasitic capacitance (leakage current) and electrochemical reaction, maintaining transfer characteristics.

The optical microscopy (OM) image of R‐EDLT is presented on the lower side of Figure 1a. Figure 1b and Figure S2a, Supporting Information, show the transfer and output characteristics of R‐EDLTs, respectively. The R‐EDLTs exhibited a field‐effect mobility of 54.4 cm^2^ V^−1^s^−1^ and an on/off ratio of 1.3 × 10^6^. As described, the relatively high on/off ratio compared to typical IG‐gated transistors can be attributed to the presence of a monolithic Al_2_O_3_ layer on Al electrodes.^[^ ^21^ ^]^ Moreover, the high mobility of the device indicates that sufficient charge accumulation can be induced at the channel layer even with the presence of the SU‐8 layer, owing to the high capacitance of EDL formed in the IG gate dielectric layer and the carrier diffusion over the offset as we mentioned above.^[^ ^27^, ^28^ ^]^ Also, the frequency‐dependent capacitance variation of IG gate dielectric is examined and presented in Figure S1b, Supporting Information. The IG gate dielectric showed a gradual reduction of capacitance value as typically observed in IG‐based gate dielectric films.

In an ideal case such as two conductive surfaces containing ionic liquid like a capacitor structure, the potential drop in the IG gate dielectric mainly occurs only at the interfaces of gate electrode/IG and *a*‐IGZO/IG layers. However, with the side‐gate geometry, an additional potential drop is expected in the IG gate dielectric possibly due to the undesired impurities present in the IG dielectric layer.^[^ ^29^ ^]^ As a result, the potential near the gate electrode would be higher than those regions far from the gate electrode. To investigate this phenomenon in detail, we investigated the current linearity as a function of channel width. In the saturation region, the drain current (*I_D_*) can be expressed by the following equation:

(1) $$I_{D}=\frac{1}{2}\mu_{n}C_{\mathrm{ox}}\left(\frac{W}{L}\right){\left(V_{\mathrm{GS}}-V_{\mathrm{th}}\right)}^{2}$$

where *μ_n_* is electron mobility, *C*_ox_ is dielectric capacitance, *W* and *L* are the width and length of the channel, *V*_GS_ is gate bias, and *V*_th_ is the threshold voltage. According to the equation, the drain current should be linearly proportional to the channel width. Thus, we varied the channel width of R‐EDLTs as 1000 µm (*W*_ch1_), 700 µm (*W*_ch2_), and 400 µm (*W*_ch3_) as illustrated in Figure 1c and compared the current linearity. Figure 1d shows the drain current variation with the channel width. Compared to the estimated drain currents for *W*_ch2_ and *W*_ch3_ (dotted lines), which were calculated from *W*_ch1_ by multiplying 0.7 and 0.4, respectively, the measured drain currents were relatively higher than the estimated values, showing an evidence of the unintended potential drop. To identify the actual potential drop occurring in the IG dielectric, the potentials at four separate points (P1∼P4) were measured parallel to the channel width direction as illustrated in the inset of Figure 1e. As displayed, a gradual potential drop was observed, and particularly, the potential near the gate electrode (P1∼P2) was higher than those far from the gate electrode (P3∼P4). We argue that although the device is capable of inducing EDL formation at both sides, the presence of impurities results in a non‐uniform potential profile over the channel layer, which has to be reduced not only for linear scalability but also for stable and highly sensitive electrostatic sensing devices. Consequently, this non‐linearity of R‐EDLT may be not eligible to fully satisfy the precise scalability like conventional transistors using solid‐state gate dielectrics.

**Figure 2:**
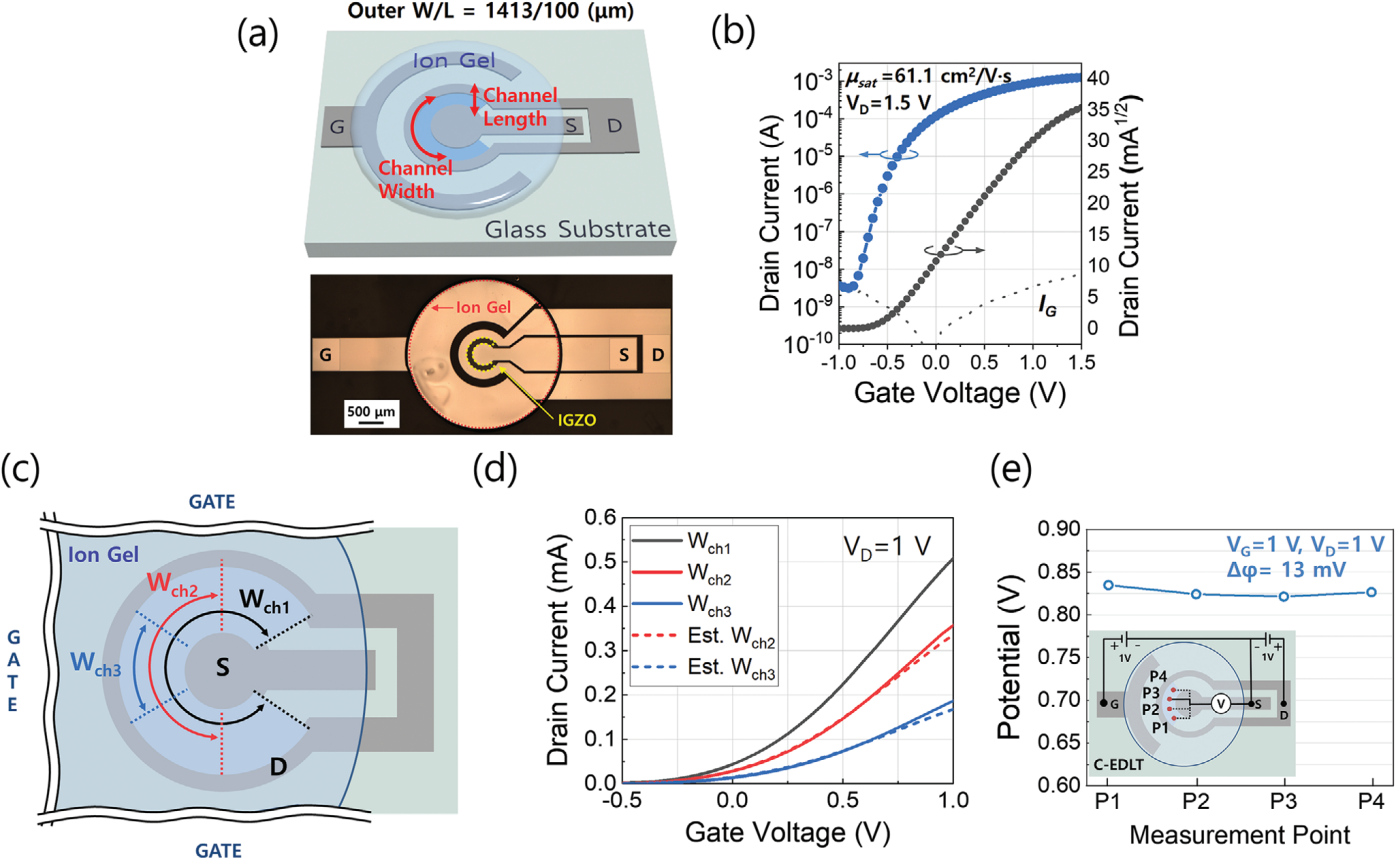
a) The device structure of a C‐EDLT and its optical microscopic (OM) image. b) A transfer characteristic of a C‐EDLT. The gate voltage was swept from −1 to 1.5 V with a step of 25 mV while the drain voltage was 1.5 V. The *V*_th_ was −0.5 V and the average saturation mobility of five different C‐EDLTs was 61.1 cm^2^ V^−1^·s ^−1^ with a SD of 2.424. The dotted line represents gate leakage current. c) Schematic diagram of the channel width variations. The widths of *W*_ch1_, *W*_ch2_, and *W*_ch3_ are 1000, 700, and 400 µm, respectively. d) Transfer characteristics of C‐EDLTs with different channel widths. The colored solid lines represent experimentally measured current characteristics. The colored dash lines represent estimated drain currents (Est. *I*_D_(*W*_ch2_) = *I*_D_(*W*_ch1_) × 0.7, Est. *I*_D_(*W*_ch3_) = *I*_D_(*W*_ch1_) × 0.4). e) The potential profile of the IG gate dielectric along the channel width direction under *V*_G_ = *V*_D_ = 1 V. Each point data are the representative of five repeated measurements with an average SD of 0.0073. The inset shows the measurement points of the potential.

As a geometrical strategy to reduce the unsymmetric potential drop along with channel width, we employed a symmetrically gated Corbino structure EDLT (C‐EDLT) in which the distance between the circular gate electrode and the channel is nearly identical as shown in **Figure** **2**a. Also, Figure S3, Supporting Information, shows the cross‐sectional structure of the C‐EDLT. Figure 2b and Figure S2b, Supporting Information, show the transfer and output characteristics of C‐EDLT, respectively, demonstrating a high field‐effect mobility of 61.1 cm^2^ V^−1^s^−1^ and an on/off ratio of 2.79 × 10^6^.

To evaluate the variation of drain current with the channel width in C‐EDLT, the channel width was varied as 1413 µm (*W*_ch1_), 942 µm (*W*_ch2_), and 471 µm (*W*_ch3_) (Figure 2c), and their transfer characteristics were measured. Also, the estimated drain current for *W*_ch2_ and *W*_ch3_ (dotted lines) were calculated from *W*_ch1_ by multiplying 0.66 and 0.33, respectively (Figure 2d). Although there were small differences at high *V*_G_, significantly improved current linearity was observed compared to the R‐EDLT. Likewise, we measured the potential drop profile parallel to the channel width direction that was the same direction for R‐EDLT as depicted in the inset of Figure 2e. In contrast to the gradual potential drop in R‐EDLT, the potential profile of C‐EDLT shows consistency as shown in Figure 2e. Although the small potential drop was also observed in the direction from gate to channel area (perpendicular to the width direction) in C‐EDLT, C‐EDLT exhibits much uniform potential distribution along channel width than those of R‐EDLT due to the symmetric distance between the circular gate electrode and the active layer. To translate these device structures in on‐chip device applications, scaled‐down devices are also fabricated, and the device performance and degree of potential drop in both devices are compared in Figures S4 and S5, Supporting Information, respectively. As shown in these figures, the 70% scaled‐down device (W/L = 300/30 µm) exhibits almost similar device performance such as field‐effective mobility and drain current. Meanwhile, since the unexpected potential drop mainly comes from the non‐ideal ionic dielectric and the asymmetric gate field over the channel layer, and as expected, the scaled‐down device can somewhat relieve the undesirable inhomogeneous potential distribution. For more efficient ion‐sensing applications, however, an enlarged interaction area with the target species is often needed and thus, some amount of potential drop along with channel width of R‐EDLT seems to be inevitable.

**Figure 3:**
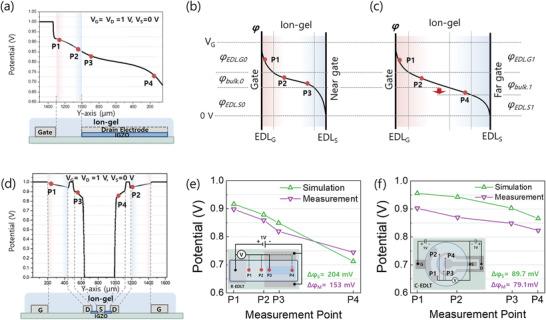
a) The potential distribution of FEA simulation over the channel region of R‐EDLT with *V*_G_ = *V*_D_ = 1 V and *V*_S_ = 0 V. Schematic diagrams of potential distribution b) near region and c) far region from the gate electrode. (EDL_G_ and EDL_S_ refer to the EDLs formed at the gate electrode and semiconductor layer, responsively). d) The potential distribution of FEA simulation over the channel region of C‐EDLT with *V*_G_ = *V*_D_ = 1 and *V*_S_ = 0 V. e) Potential profiles along with the channel width direction for R‐EDLT from FEA simulations and measured values. Each measured point data are the representative of five repeated measurements with an average SD of 0.0043. f) Potential profiles along with the channel width direction for C‐EDLT from FEA simulations and measured values. Each measured point data are the representative of five repeated measurements with an average SD of 0.0031.

For a more in‐depth understanding of the potential drop occurring in the IG dielectric, we carried out a computational simulation based on the finite element analysis (FEA). **Figure** **3**a shows the simulation results for the variation of potential under *V*_G_ = 1 V and *V*_D_ = 1 V, which were extracted from the channel (IGZO) surface (center between source and drain electrodes). In this case, gradual potential drops were observed which are in good agreement with the experimental results. Figure 3b,c shows the possible models for the potential variation over the near gate and far gate region, respectively. When *V*_G_ is applied to the gate electrode, EDLs are formed at each interface where the potential drops dominantly occur. Particularly, the gate potential is decreased by *φ_EDL.G0_* and *φ_EDL.S0_* at the gate electrode and at the semiconductor surface region, respectively. In this case, *φ_EDL.S0_* is slightly less than or similar to *φ_EDL.G0_* (*φ_EDL.S0_* ≤ *φ_EDL.G0_*) due to the different polarization effect with the metal/IG and semiconductor/IG interfaces, respectively.^[^ ^30^, ^31^, ^32^, ^33^ ^]^ More importantly, in the bulk region of IG dielectric, a gradual potential decrease has also occurred (*φ_bulk.0_*). Therefore, at relatively far gate regions, the potential drop (*φ_bulk.1_*) in the IG dielectric becomes larger than *φ_bulk.0_* due to the longer distance from the gate electrode, leading to a decreased potential as depicted in Figure 1e. In an ideal case, potential drops occurred only at EDLs, accompanying homogenous charge accumulation along with entire channel width due to identical gate field from the gate electrode to channel area. However, there is a parasitic potential drop within the ionic dielectrics due to undesired impurities. Therefore, a linearly decreased potential drop can appear along with the channel width direction, generating a non‐uniform charge accumulation, which consequently leads to non‐linear drain current as a function of channel width (Figure 1d). In the C‐EDLT, even though the gradual potential drop can occur similar to the R‐EDLT, a symmetric potential profile was observed due to the constant distance between the gate electrode and IGZO surface. Figure 3d shows the simulation results for the potential variations in the C‐EDLT, which were extracted from the channel (IGZO) surface (center between source and drain electrodes). Figure 3e,f demonstrates the position (P1, P2, P3, and P4)‐dependent potential profiles of R‐EDLT and C‐EDLTs, respectively. As depicted in the figures, simulation and measurement results are in good agreement, validating the aforementioned underlying physics. Although a potential drop is observed between the gate electrode and IGZO layer, the potential profile is symmetric alongside the channel width. Since the non‐linear scalability is more important over the IGZO channel layer for the EDLT applications, we also verified the potential profiles of the simulation and measurement results over the channel region. As plotted in Figure S6, Supporting Information, potential profiles of C‐EDLTs exhibit much uniform characteristics in both simulation and measurement results.

To further investigate the potential distribution in the entire device area, 3D simulations were carried out for various bias conditions. Figures S8 and S9, Supporting Information, show the corresponding simulation results for R‐EDLT and C‐EDLT, respectively. They show the corresponding simulation results for *V*_G_ = 1 V (*V*_S_ = 0 V, *V*_D_ = 1 V). Since the S/D electrodes are passivated by the thick SU‐8 photoresist (≈3 µm), the potential variation from undesired ion‐to‐electron conversion between IG and S/D electrodes can be effectively suppressed, delivering no field effect to the IG. More importantly, the thick polymer passivation blocks the electric field between the S/D electrode as well as the gate and S/D as we reported in our previous study.^[^ ^21^ ^]^ At the far gate region in Figure S8, Supporting Information, a relatively low potential level with potential drop was observed over the channel region. This can be attributed to the lower applied gate voltage at points far away from the gate, resulting in a relatively lower charge accumulation effect at the semiconductor surface as depicted in Figure 3c.

**Figure 4:**
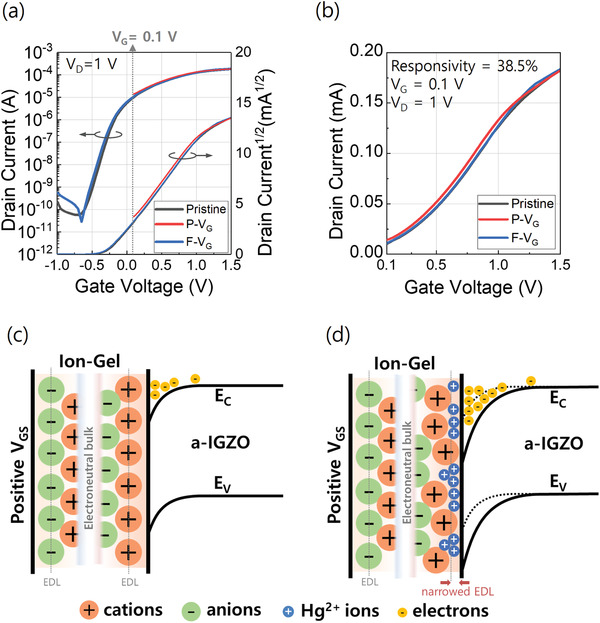
Transfer characteristics of R‐EDLT with a) full scale and b) positive scale of *V*_G_. Black, red, and blue solid lines represent pristine, positive *V*_G_ sweep (P‐V_G_), and full *V*_G_ sweep (F‐V_G_) transfer characteristics, respectively. Illustrations of the ion sensing mechanism for c) pristine operation state, and d) sensing operation state of under positive *V*_G._ The smaller Hg^2+^ ions are interstitially dispersed closer to the interface compared to the cations, narrowing the EDL width and attracting more electrons at the interface.

Although the EDLTs exhibits unintended potential drop, such ionic dynamics can offer considerable potential to be utilized as ion sensing applications. Accordingly, we investigated the ion sensing characteristics of R‐EDLT and C‐EDLT using mercury ions (H_g_^2+^) as the target analyte. Over the IG dielectric, a micro‐droplet of mercury ion solution (5 ppm, 0.3 µL) was dropped and left for around 3 min to fully absorb into the IG film. **Figure** **4**a,b shows the corresponding transfer characteristics of R‐EDLT. Compared to the pristine device (black solid lines), a small negative shift was observed with the presence of H_g_^2+^ ions (red solid lines). Here, only positive *V*_G_ was applied (P–*V*_G_ sweep, +0.1 V → +1.5 V) to move the H_g_^2+^ ions toward the interface between the IG dielectric and *a*‐IGZO semiconductor layer. The R‐EDLT exhibited a low responsivity of 38.5% which was calculated by the equation:

(2) $$\mathrm{Responsivity}\left(\%\right)=\frac{I_{S}-I_{0}}{I_{0}}\times 100$$

where *I_0_* is the initial current and *I_S_* is the current after sensing. As described, we observed that the shift of the transfer curve was very small even with the presence of H_g_^2+^ ions and the responsivity became almost zero with increasing *V*_G_ (*V*_G_ > 1 V) as shown in Figure S10, Supporting Information. Such sensing characteristics of the device can be explained by the models shown in Figure 4c,d.^[^ ^31^ ^]^ Initially, when the analyte solution was dropped over the IG surface, the H_g_^2+^ ions electrostatically attract opposite charges in the IG dielectric. Then, an EDL is formed with charges of a solid surface. During this process, unexpected electrochemical reactions might occur which provide additional electrons to the surface. This charge electrification mechanism can be also applied to the IG/semiconductor interface.^[^ ^34^ ^]^ The diameter of EMIM cation is about 1 nm, and the atomic diameter of Hg^2+^ is about 0.220 nm.^[^ ^35^ ^]^ Therefore, the smaller Hg^2+^ ions can be interstitially dispersed more close to the interface and may electrostatically couple with the electrons in *a*‐IGZO without an external voltage, resulting in subtle EDL capacitance. With an external voltage, relatively smaller Hg^2+^ ions can be more densely occupied near the *a*‐IGZO surface, being surrounded by relatively large EMIM cations. Consequently, the H_g_^2+^ ion concentration is increased at the interface between IG and *a*‐IGZO, by which more electrons are accumulated in the *a*‐IGZO layer as shown in Figure 4d (slightly narrower EDL than before), and results in a higher current level compared to the pristine device. Meanwhile, under the higher gate bias (*V*_G_ > 1 V), given that a large external voltage induces ion crowding at the surface,^[^ ^36^ ^]^ capacitive coupling of EMIM cations and electrons at the interface become dominant, diminishing the Hg^2+^ ions‐induced current and responsivity to the Hg^2+^ ions. Interestingly, after a full *V*_G_ sweep (*F*–*V*_G_ sweep, −1 V → +1.5 V), the transfer curve was recovered like the pristine characteristics as shown in Figure 4a,b (blue solid lines). Presumably, the rearrangement of ion stacking with reverse gate bias may compensate the intensified EDL capacitance by the interstitial Hg^2+^ ions, which can be a beneficial route to achieve reusable sensor electronics.

**Figure 5:**
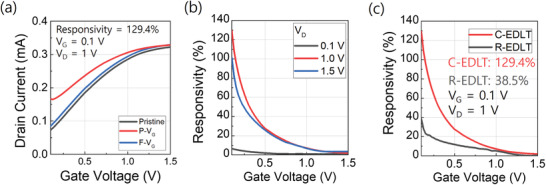
a) Transfer characteristics of C‐EDLT during ion sensing. b) Responsivity as a function of *V*_G_. The *V*_D_ was varied as 0.1 V (black solid), 1.0 V (red solid), and 1.5 V (blue solid). The responsivity values were 6.4%, 129.4%, and 101.3%, respectively. c) Responsivity as a function of *V*_G_ for C‐EDLT (red solid) and R‐EDLT (black sold).

According to the ion dynamics of the sensing mechanism, the Corbino structure enables the ions and external analytes to be symmetrically distributed over the channel layer, by which the target ions can interact with the channel symmetrically, resulting in more uniform sensing characteristics compared to the R‐EDLT. As shown in **Figure** **5**a, P–*V*_G_ and F–*V*_G_ sweeps were performed for C‐EDLT, and under the P–*V*_G_ sweep, the C‐EDLT exhibited a high responsivity of 129.4% to H_g_^2+^ ions. Further to enhance the responsivity, we optimized the V_D_. Figure 5b shows the corresponding results and the maximum responsivities were 6.4%, 129.4%, and 101.3% for *V*_D_ of 0.1, 1.0, and 1.5 V, respectively (*V*_G_ = 0.1 V). The highest responsivity was observed at *V*_G_ = 0.1 V and *V*_D_ = 1.0 V, which indicates that a low VG with a moderate VD for withdrawing electrons and a low *V*_G_ enabled minute sensing signal over the low concentration of external analyte to be significantly emerged. The highest responsivity achieved with rather moderate *V*_D_ ( = 1.0 V) can be understood by the electrostatic interaction between analytes and induced charges on the surface of IGZO channel layer. More depleted channel region can be generated by the higher *V*_D_ ( = 1.5 V), where fewer electrons to interact with the analytes are existed at the channel surface area, leading to lower responsivity at the higher drain bias. The responsivity characteristics of R‐EDLT and C‐EDLT are summarized in Figure 5c, which clearly indicates that the responsivity was significantly enhanced by using the C‐EDLT (more than three times). In contrast to the large potential variation occurring in the R‐EDLT, the EDL capacitance of gate/IG/channel in the C‐EDLT is almost identical over the whole region. As a result, more Hg^2+^ ions can be symmetrically reached onto the whole channel surface, inducing a larger density of counterpart electrons. Finally, to clarify that the sensing signal came from the excessive electrostatic coupling between the Hg^2+^ ions and electrons, we compared the transfer curves before and after sensing a droplet of deionized water (DIW). As shown in Figure S11, Supporting Information, there was no significant increase of drain current at *V*_G_ = 0.1 V, which implies that the Hg^2+^ ions dominate the change of drain current at low bias conditions.

## 3 Conclusion

In conclusion, we demonstrated an EDLT based ion‐gated in‐plane EDLT as a basic building block for integrated bioelectronics. In particular, by adopting a symmetrically gated electrode structure in the EDLT, a high ion sensitivity could be achieved which was more than 3 times higher than the R‐EDLT. Furthermore, we carried out the computational simulation to investigate the potential distribution in EDLTs depending on the electrode structure and found out the current linearity could be improved by using the novel structure. From these findings, we envision that the proposed C‐EDLT can be a promising candidate for a basic building block in integrated bioelectronics.

## 4 Experimental Section

### IG Solution Preparation

1‐ethyl‐3‐methylimidazolium bis(trifluoromethylsulfonyl)imide (EMIM‐TFSI), poly(ethylene glycol) diacrylate (PEGDA), and 2‐hydroxy‐2‐methyl‐propiophenone (HOMPP) were used for the fabrication of IG films. EMIM‐TFSI and PEGDA were mixed with a volume ratio of 8:2, and then, HOMPP was added to the mixed solution with a volume concentration of 3%. The final solution was stirred at 80 °C for 6 h. All the chemical substances were purchased from Merck and used as received.

### Device Fabrication

A glass substrate was first cleaned by sonicating in acetone and isopropyl alcohol for 10 min, respectively. To remove any undesired organic residues and to form a hydrophilic surface, oxygen plasma treatment was carried out using a reactive ion etching system. The cleaned glass substrate was thermally treated at 200 °C for 5 min. Then, a 30 nm‐thick *a*‐IGZO film was deposited on the glass substrate by RF sputtering using an IGZO target (In:Ga:Zn = 1:1:1 mol%). Afterward, the sample was thermally annealed at 300 °C for 1 h and patterned by using conventional photolithography processes. To fabricate the EDLT devices with a side‐gate geometry, an undercut layer was fabricated using a negative photoresist (NR9‐3000PY, Futurrex), and a 100‐nm‐thick Al layer was deposited via a thermal evaporator. Subsequently, to form a monolithic Al_2_O_3_ layer, UV irradiation was conducted for 30 min with a mercury lamp under ozone flow generated by a built‐in ozone generator (UV‐1, SAMCO, emission wavelengths of 253.7 and 184.9 nm). 99.95% of pure oxygen gas was continuously injected with a flow rate of 0.1 L min^–1^. Then, the Al electrode was lifted off in acetone and thermally treated at 150 °C for 1 min. Additionally, ≈3‐µm‐thick SU‐8 layer (SU8‐3005, MicroChem) was deposited by using a spin coater (4500 rpm, 30 s), which is thermally annealed at 95 °C for 1 min. The sample was exposed to UV with a chrome mask for 8 s (120 mJ cm^–2^), and two steps of the thermal annealing were orderly carried out for 65 °C for 1 min and 95 °C for 1 min, respectively. The unexposed region of SU‐8 film was developed for 1 min with strong agitation, and the sample was rinsed with IPA. Consequently, the 3‐µm‐thick of SU‐8 bank was formed, revealing the surface of IGZO film and gate electrode of the photo‐assisted AlO_X_/Al only. Lastly, to form an IG dielectric on selective locations, the IG solution was drop‐casted onto the side gate device, and a UV exposure was carried out with a photomask for 30 s. The device was rinsed with DIW and dried with nitrogen to remove residue IG solution. The width and length of the rectangular geometry (rectangular EDLT: R‐EDLT) are 1000 and 100 µm, respectively. For Corbino geometry (Corbino EDLT: C‐EDLT), the outer and inner widths are 1413 and 942 µm, respectively, while the length is 100 µm.

### Characterization

All the electrical measurements were carried out in a dark box at room temperature with a parameter analyzer (4156C, Agilent) for EDLT devices including the inverter circuit and an LCR meter (4284A, Agilent) for capacitance values. The surface morphology of the metal oxide film was analyzed with an atomic force microscopy (NX‐10, Park systems). In the potential measurement, direct contact to the surface of the target layer (at the points of P1∼P3 and P4) throughout the ion‐gel (IG) and pre‐defined (inserted) inner electrodes (Figure S7a,b, Supporting Information) in the device structure was employed with an Au‐coated probe tip. The measured potential values exhibit negligible differences by the measurement types, validating both measurement methods. (Figure S7c,d, Supporting Information).

### FEA Multiphysics Simulation

The simulation was performed to help understand the formation of an electric double layer (EDL) at the interface between electrode and electrolyte. EDL was composed of a stern layer and a diffuse layer, and the Gouy–Champman–Stern model is applied to COMSOL Multiphysics. By analyzing the Nernst‐Plank equation connected with the Poisson equation, 3D finite element modeling was carried out by applying the same size as the actual device. More detailed descriptions are provided in the Supporting Information.

### Statistical Analysis

Five EDLTs for each structure type (Corbino and rectangular) and size (W/L: 1000/100 um and 300/30 um) were fabricated and characterized for their transfer properties throughout the manuscript. The representative curves were presented with the average mobility and standard deviation for each case. Additionally, potential profiles were also repeatedly measured for five times in each case, presenting the representative point data and an average of the standard deviations for each measurement point. Calculation of the statistical data was carried out using EXCEL (Office 365, Microsoft Corporation, USA) and data analysis through Origin (Origin lab Corporation, USA).

## Conflict of Interest

The authors declare no conflict of interest.

## Supporting information

Supporting Information

## Data Availability

The data that support the findings of this study are available from the corresponding author upon reasonable request.
