# Supplementary material for: Symmetrically Ion‐Gated In‐Plane Metal‐Oxide Transistors for Highly Sensitive and Low‐Voltage Driven Bioelectronics
Source: Adv Sci (Weinh). 2022 Mar 3;9(13):2103275. doi: 10.1002/advs.202103275 (PMC9069198; doi:10.1002/advs.202103275)
Supplement: Supplementary file 1 — Supporting Information [file ADVS-9-2103275-s001.pdf]

Supporting Information

Symmetrically Ion-Gated In-Plane Metal-Oxide Transistors for Highly Sensitive and Low-Voltage Driven Bioelectronics

Jingu Kang[1†], *Young-Woo Jang*[1†], *Sang Hee Moon* [1], *Youngjin Kang*[2], *Jaehyun Kim* [3], *Yong-Hoon Kim* [2]*, *and Sung Kyu Park* [1]*

[1] School of Electrical and Electronics Engineering, Chung-Ang University, Seoul 06974, Korea

[2] School of Advanced Materials Science and Engineering, Sungkyunkwan University, Suwon 16419, Korea

[3] Department of Chemistry and Materials Research Center, Northwestern University, 2145 Sheridan Road, Evanston, IL 60208, USA

† J. K. and Y. J. contributed equally to this work.

Corresponding Author: Prof. Sung Kyu Park (skpark@cau.ac.kr) and Prof. Yong-Hoon Kim (yhkim76@skku.edu)

This "Supporting Information" includes:

Figure S1 to S6, Table S1

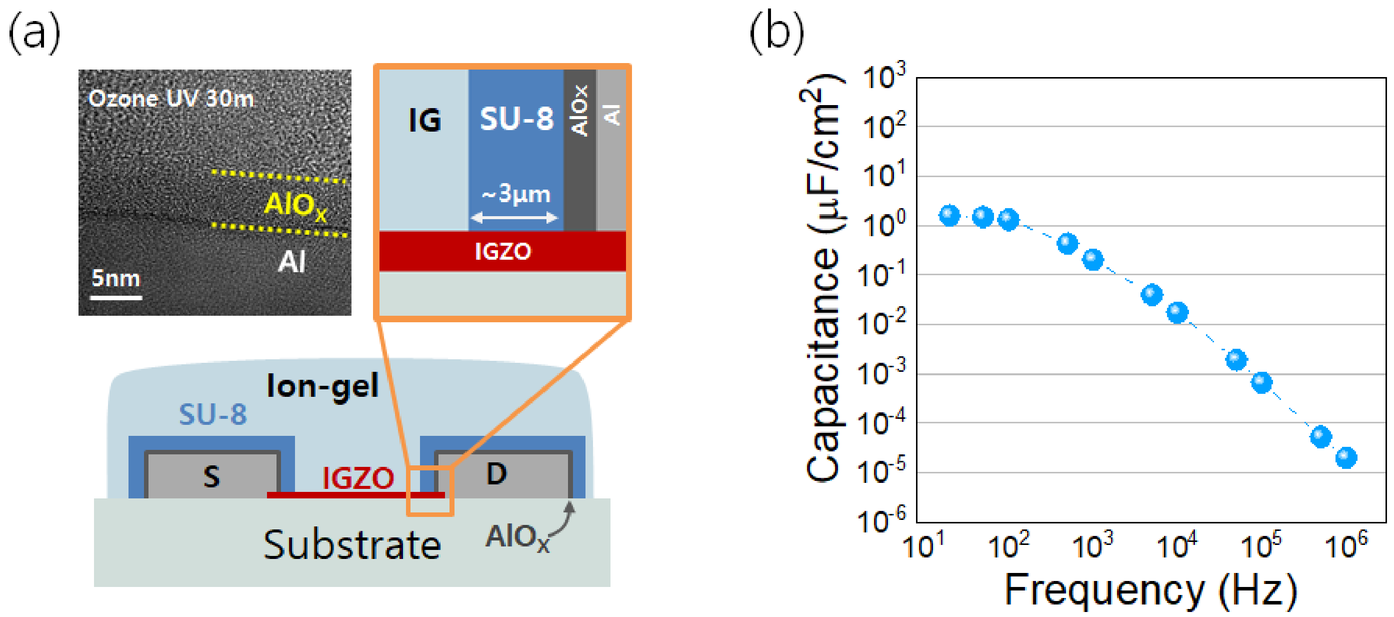

Figure S1. a) A schematic and SEM image (inset) of the side view of a R-EDLT. b) Frequency vs. capacitance of the ion gel dielectric.

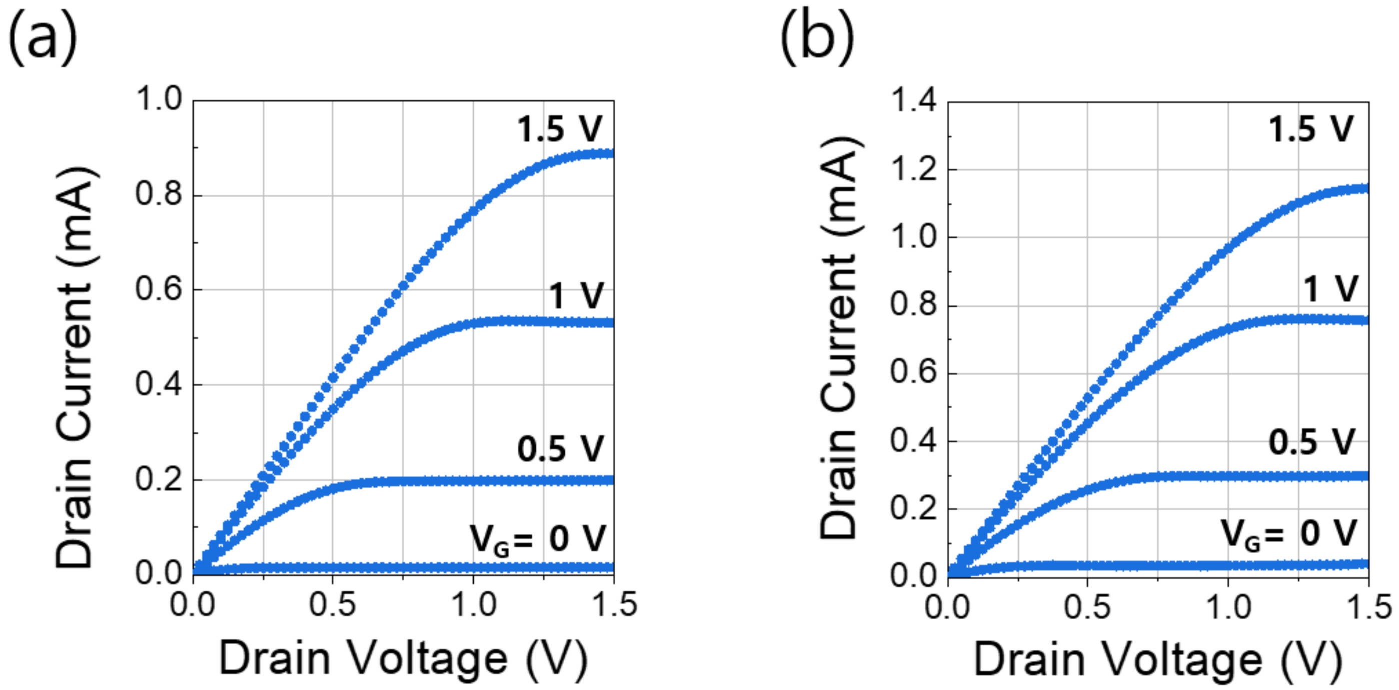

Figure S2. Output characteristics of the a) R-EDLT and b) C-EDLT. V_D was swept from 0 V to 1.5 V with 0.5 V step of V_G.

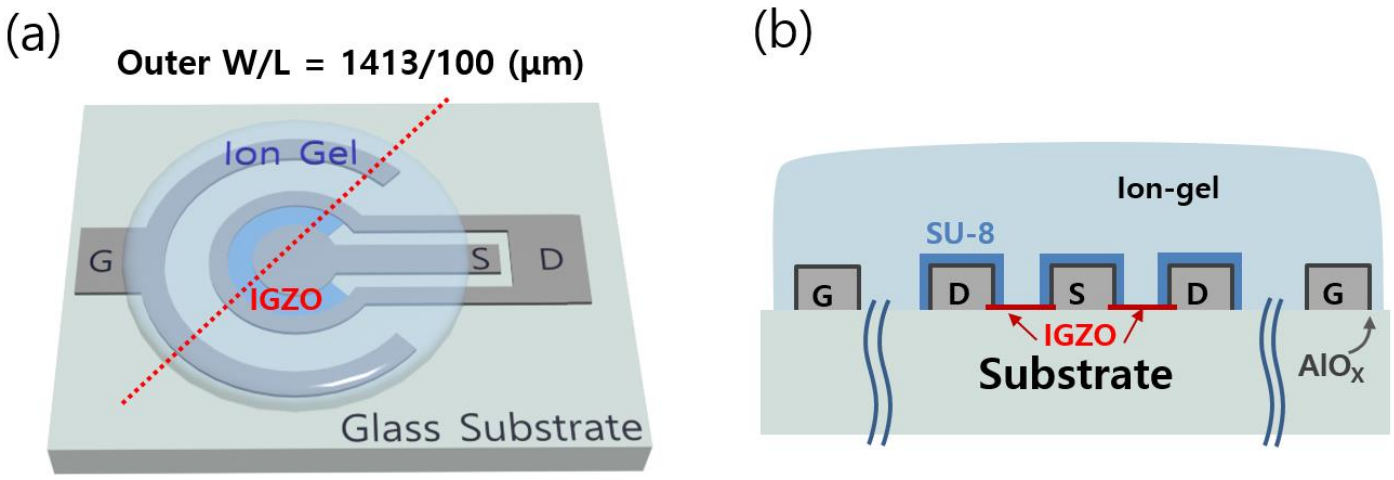

Figure S3. a) The device structure of a C-EDLT. b) A schematic image of the side view of a C-EDLT.

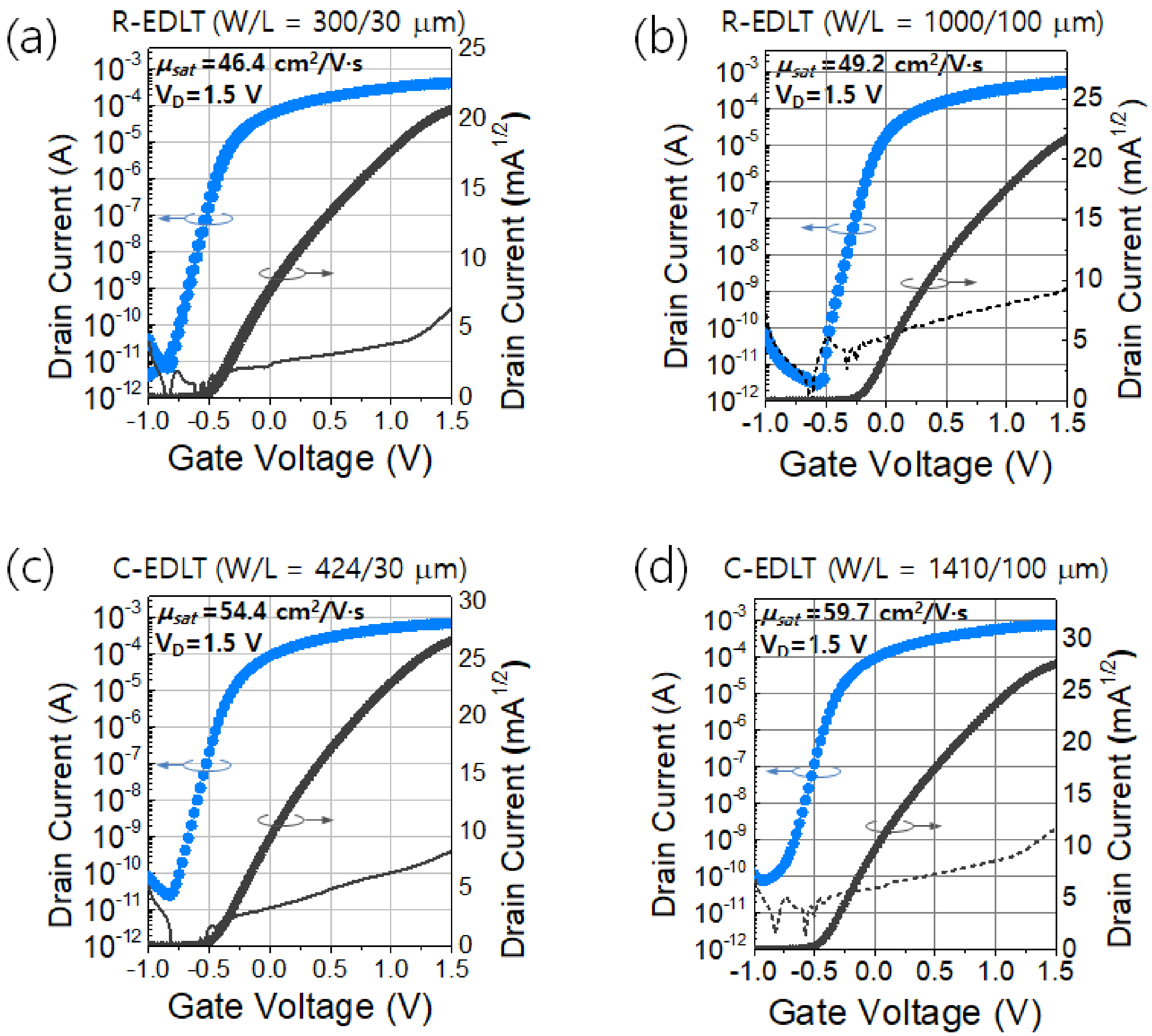

Figure S4. The transfer characteristics of a R-EDLT with widths and lengths of a) 300 μm, 30 μm and b) 1000 μm, 100 μm, respectively. The small-scaled R-EDLTs had an average mobility of 50.6 $cm^2\ V^{-1}s^{-1}$ with a SD of 0.85. The transfer characteristics of a C-EDLT with widths (outer) and lengths of c) 424 μm, 30 μm and d) 1410 μm, 100 μm, respectively. The small-scaled R-EDLTs had an average mobility of 56.1 $cm^2\ V^{-1}s^{-1}$ with a SD of 0.91. The dotted lines represent gate leakage current in all figures.

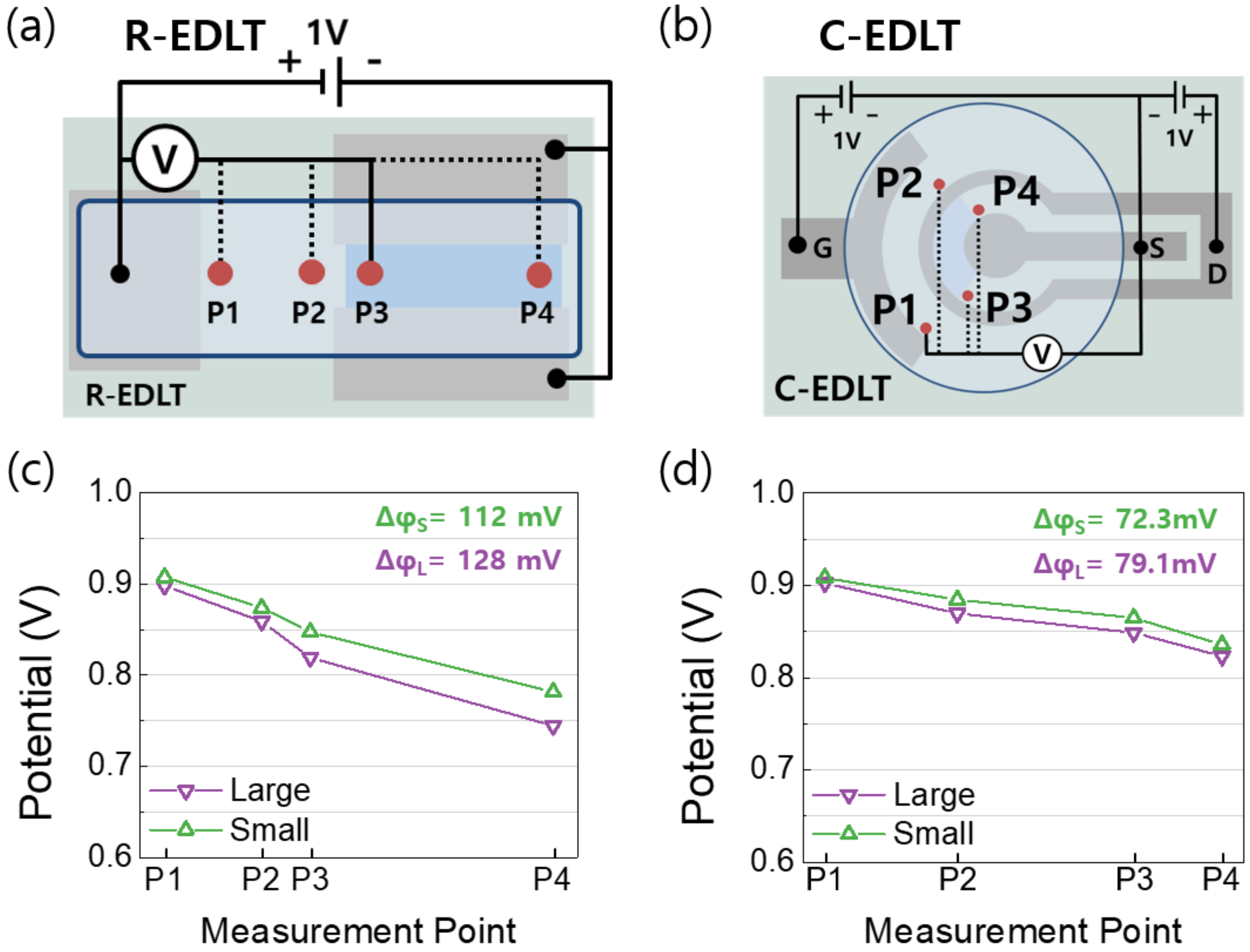

Figure S5. Measurement points of the potential throughout the IG gate dielectric in the a) R-EDLT and b) C-EDLT. Comparison of the potential profiles of the IG gate dielectric in the large and small scaled devices of c) R-EDLT and d) C-EDLT. Each point data for the small-scaled R-EDLT and C-EDLT are the representative of 5 repeated measurements with an average SD of 0.0028 and 0.0043, respectively.

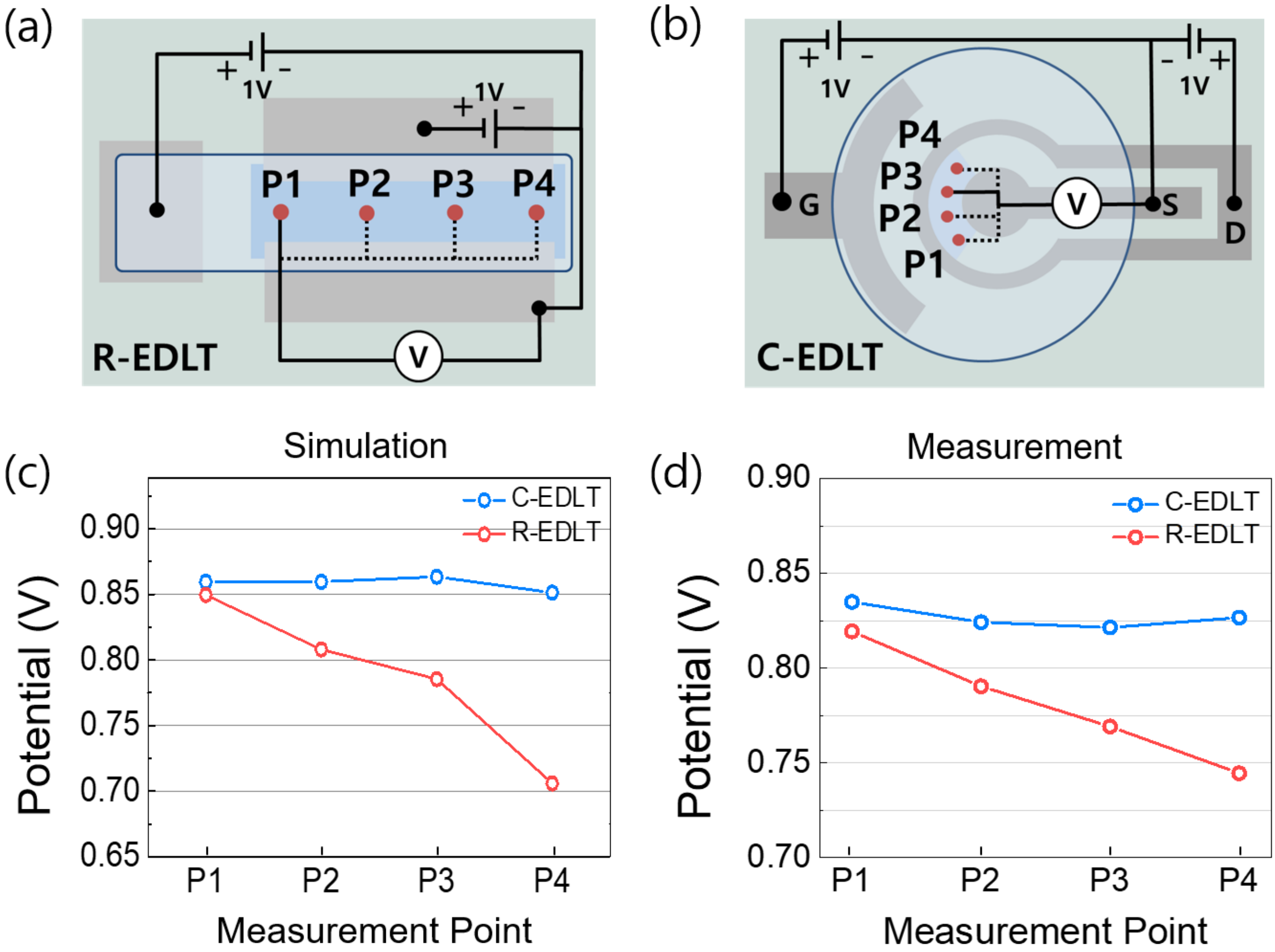

Figure S6. Measurement points of the IG gate dielectric potential along with the channel width in a) R-EDLT and b) C-EDLT. c) FEA simulation and d) measured values of the potential profile in the measurement points of the R-EDLT and C-EDLT. Each point data for the R-EDLT and C-EDLT are the representative of 5 repeated measurements with an average SD of 0.0039 and 0.0073, respectively.

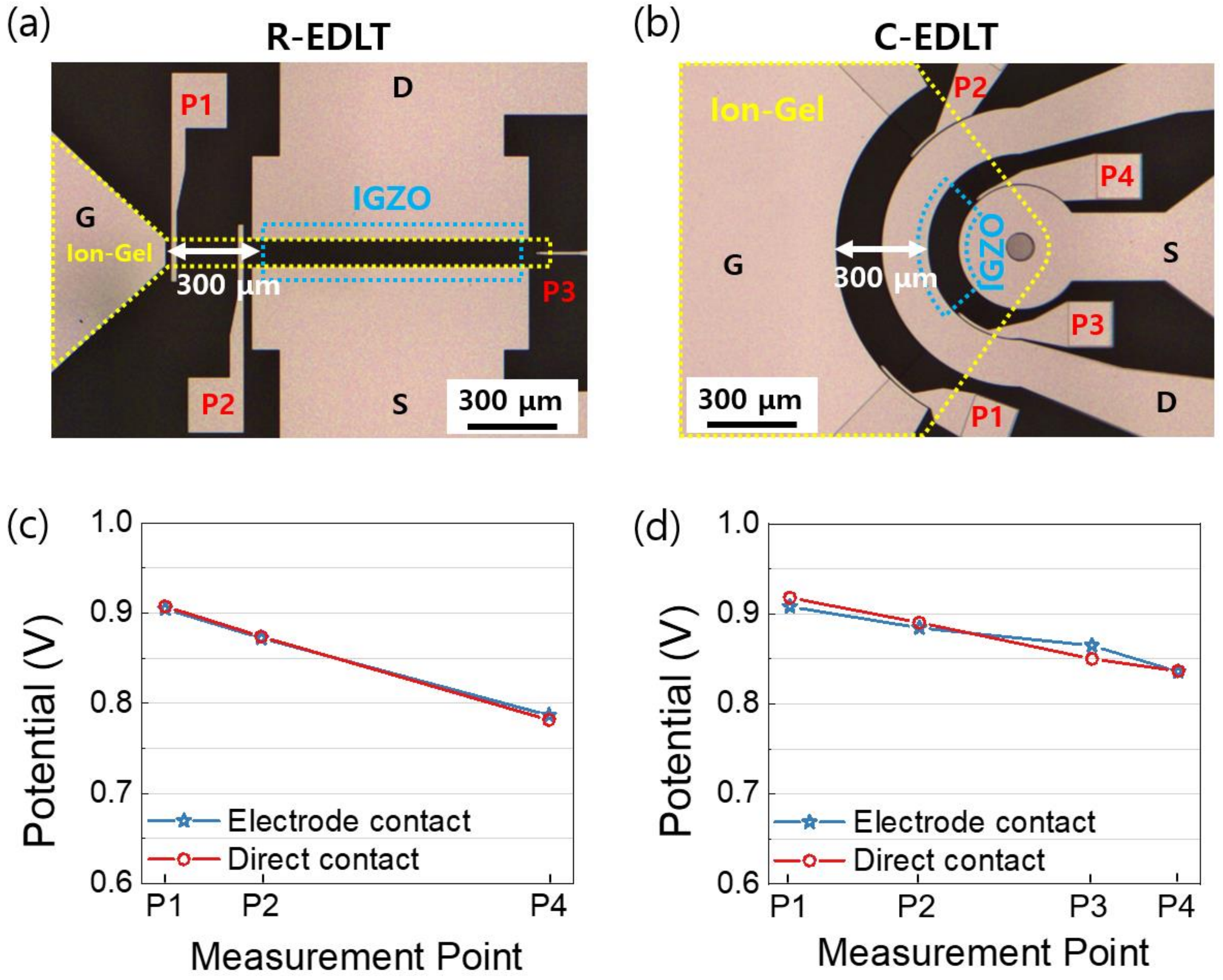

Figure S7. Optical microscopic image of the a) R-EDLT and b) C-EDLT with pre-defined inner electrodes for potential measurements. c) Comparison of the potential profiles measured by the inner electrode contact and direct contact to the target surface with probe tips in c) R-EDLT and d) C-EDLT. Each point data of the electrode contact pad measurements for R-EDLT and C-EDLT are the representative of 5 repeated measurements with an average SD of 0.0039 and 0.0076, respectively.

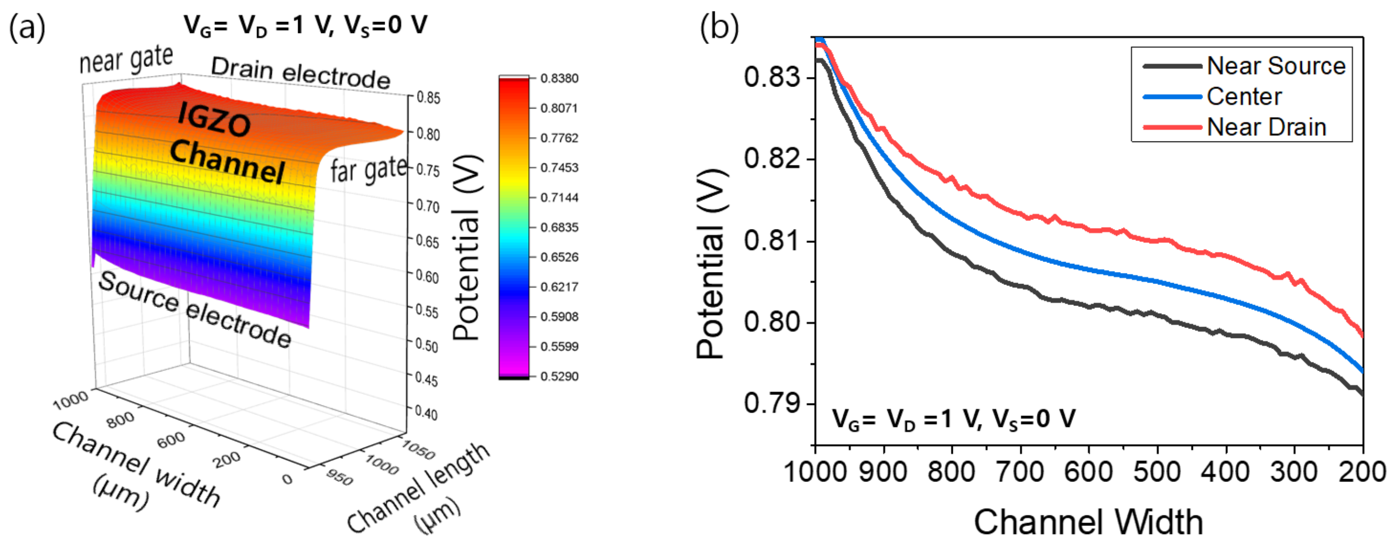

Figure S8. a) 3-dimensional simulation results of potential distribution of the R-EDLT for V_G = 1 V (V_S = 0V, V_D = 1V). b) Potential distributions alongside the channel width in 3 cases (near the source electrode, center of the channel, and near the drain electrode).

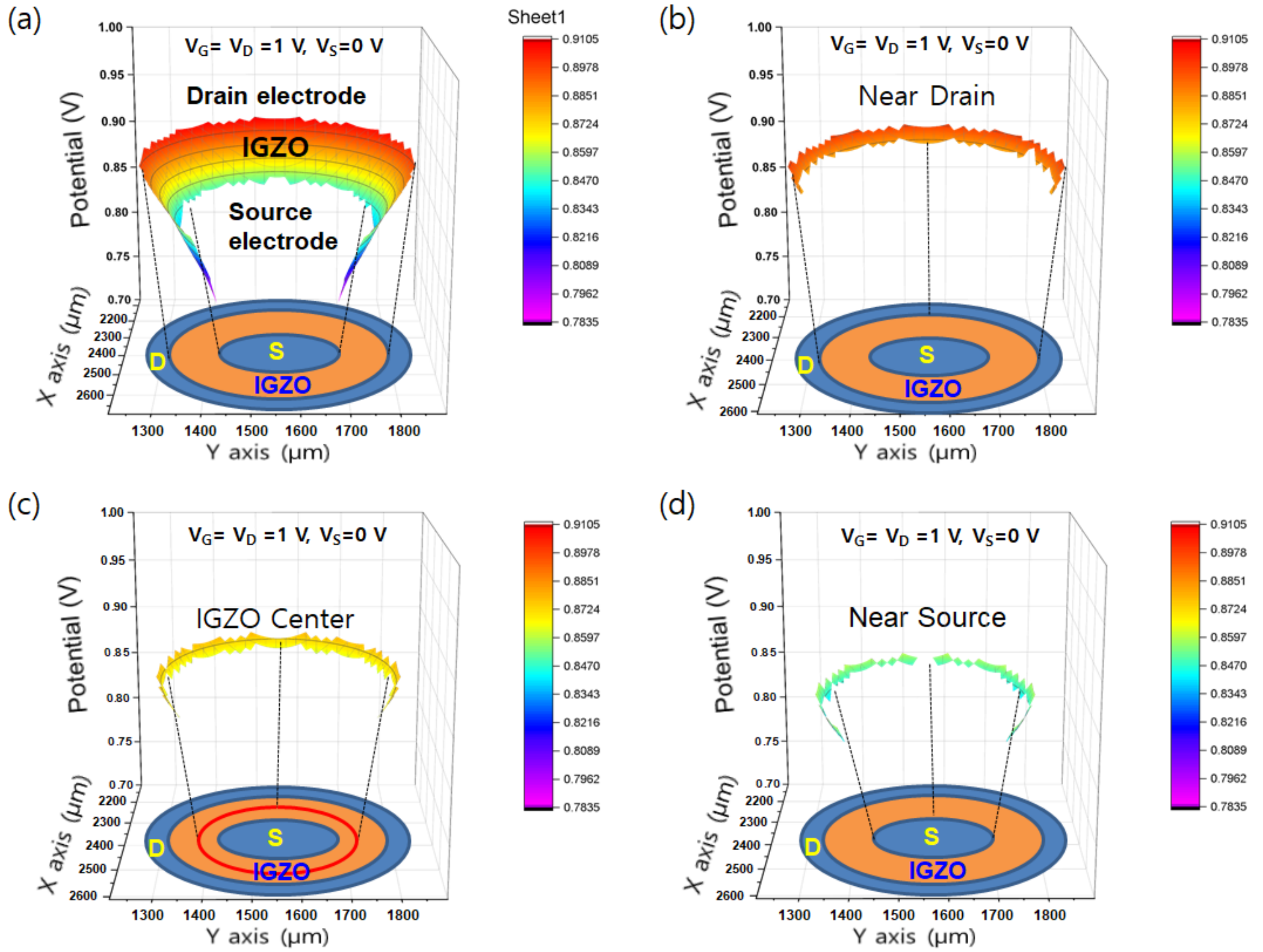

Figure S9. a) 3-dimensional simulation results of potential distribution of the C-EDLT for V_G = 1 V (V_S = 0V, V_D = 1V). b) Potential distributions of the IGZO layer that is b) near the drain electrode, c) center of the channel, and d) near the source electrode.

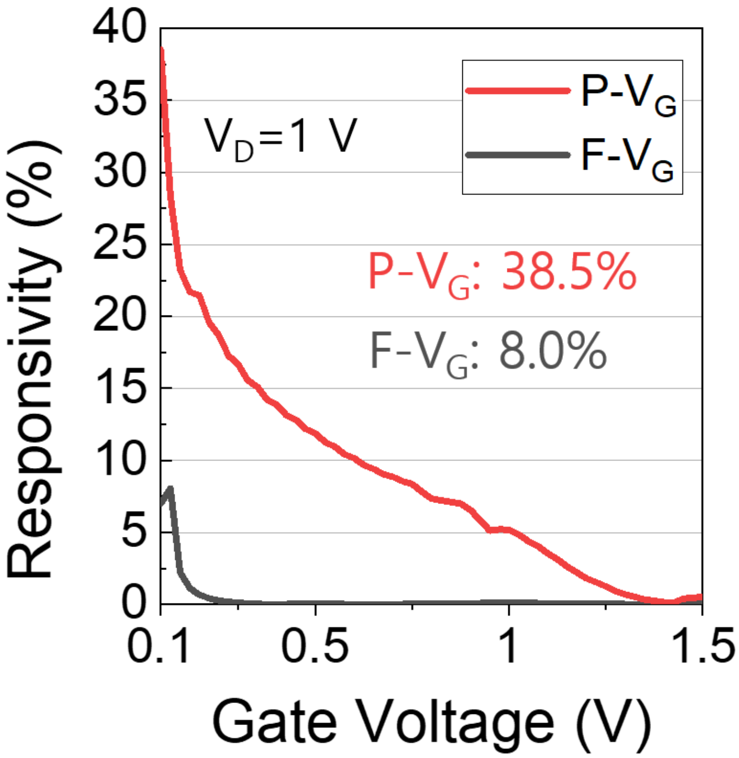

Figure S10. Gate voltage dependent responsivity variation the R-EDLT

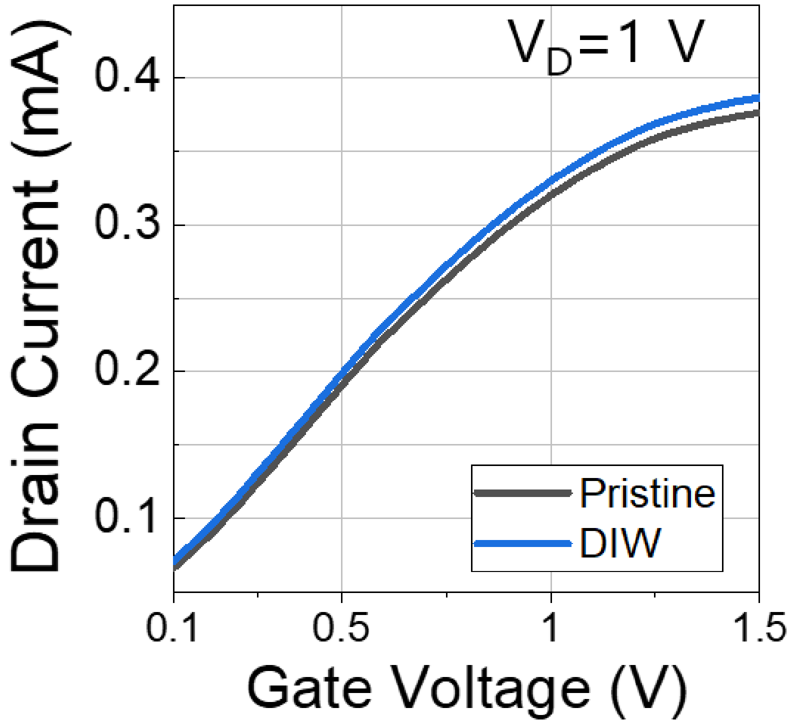

Figure S11. Transfer curves of the C-EDLT with a droplet of deionized water (DIW).

Table S1. FEA multiphysics simulation

FEA multiphysics simulation was performed to help understand the formation of electric double layer (EDL) at the interface between electrode and electrolyte. EDL is composed stern layer and a diffuse layer, and the Gouy-Champman-stern model is applied to COMSOL Multiphysics. By analyzing the Nernst-Plank equation connected with Poisson equation, 3-dimensional Finite Element Modeling was carried out by applying the same size as the actual device. Mesh is max. 2.5 μm to be implemented between the thick ion-gel (thickness 100um) and the relatively thin electrode (thickness 1um). As shown in the table S1, voltages were applied to the gate, source, and drain electrode according to the desired value, and the cations were set to concentrate on the source electrode and anions were set on the gate electrode. The electrode relative permittivity was 1, ion-gel was 16, and a-IGZO was 15.15. the charge concentration of cation and anion were each 1 mol/m^3 for modeling. The same value was applied the corbino-type.

Potential[V]	gate	source	drain
Drain current	0 ~ 1	0	1
IG dielectric	1	0	1
bias V_D effects	0.1, 1	0	1

Materials	electrode	Ion-gel	a-IGZO
relative permittivity	1	16	15.15

Table S1. FEA multiphysics simulation parameters used throughout the research
